# NatureKG: an ontology and knowledge graph for nature finance with a Text2Cypher application

**DOI:** 10.3389/frai.2025.1693843

**Published:** 2025-12-04

**Authors:** Neetu Kushwaha, Alok Singh, Hassan Aftab Sheikh

**Affiliations:** 1 Smith School of Enterprise and the Environment, University of Oxford, Oxford, United Kingdom; 2 NatureMind AI, Oxford, United Kingdom; 3 Data Science and AI in Creative Industry, Norwich University of the Arts, Norwich, United Kingdom

**Keywords:** nature finance, knowledge graphs, Text2Cypher, large language models, cypher query, Neo4j, question answering, graph database

## Abstract

**Introduction:**

Nature finance involves complex, multi-dimensional challenges that require analytical frameworks to assess risks, impacts, dependencies, and systemic resilience. Existing financial systems lack structured tools to map dependencies between natural capital and financial assets. To address this, we introduce NatureKG, the first ontology and instantiated knowledge graph (KG) specifically tailored to nature finance, aiming to support financial institutions in assessing environmental risks, impacts, and dependencies systematically.

**Methods:**

We designed a domain ontology grounded in ENCORE, the Science-Based Targets Network (SBTN), and peer-reviewed literature. This ontology defines entities such as Actions, Drivers of Nature Loss, Value Chains, Evidence, and Sources. The ontology was instantiated into NatureKG within Neo4j, consisting of 320 nodes and 540 relationships curated by domain experts. As a proof of concept, we constructed a Text2Cypher dataset and fine-tuned three open-source large language models (Phi-3, LLaMA-3.1-8B, and Mistral-7B) to translate natural language queries into Cypher graph queries. The models were trained and evaluated under different dataset split strategies (paraphrase, cypher-level, and generalization) using metrics such as BLEU, exact match, execution accuracy, and Macro F1 scores.

**Results:**

Phi-3 achieved the highest execution accuracy (0.21) and Macro F1 score (0.56), demonstrating better structural and reasoning capability under paraphrase and schema generalization splits. LLaMA-3.1-8B exhibited balanced performance, while Mistral-7B lagged across most metrics. The results indicate that smaller, fine-tuned models can generalize effectively in low-resource, domain-specific settings, validating the feasibility of LLM-assisted querying for nature finance.

**Discussion:**

Despite modest initial accuracy, this feasibility study establishes a baseline for integrating domain-specific ontologies with AI systems. NatureKG offers a reusable foundation for representing environmental risks, dependencies, and interventions, with potential to enhance transparency and scalability in sustainable finance decision support. Future work should expand dataset diversity, sectoral coverage beyond the built environment, and refine model reasoning through larger, domain-aligned data catalogues.

## 1 Introduction

Nature finance aims to recognize the economic value of ecosystems and emphasizes the need for corporations and financial institutions to integrate nature-related considerations into financial decision-making (Taskforce on Nature-related Financial Disclosures (TNFD), 2023). The goal is to better understand and manage their impacts and dependencies on nature, mitigate the financial risks associated with nature loss, and identify opportunities to invest in ecosystem restoration and sustainable use. Although our economy relies heavily on nature's services (Dasgupta, 2021), which are valued at over USD 100 trillion annually, biodiversity and ecosystem health continue to decline. The Global Biodiversity Framework (GBF) calls for coordinated action across all stakeholders to address this crisis (Convention on Biological Diversity, 2022), including closing the estimated USD 700 billion annual biodiversity finance gap (Deutz et al., 2020) by redirecting harmful capital flows and scaling nature-positive investments.

As ecosystems continue to deteriorate, the loss of natural capital threatens critical services, which directly affects businesses that rely on them (Dasgupta, 2021). These businesses are often recipients of finance, investment, and credit. As nature's ability to deliver services declines, associated risks ripple across financial systems, increasing institutional and systemic vulnerability. These cascading risks represent a serious form of physical risk. Understanding financial risk linked to nature dependence requires systematically identifying the drivers of nature loss that threaten financial systems, a challenge increasingly acknowledged by initiatives such as Finance for Biodiversity Foundation (2021). Financial institutions, as key intermediaries in global capital markets, have the potential to drive systemic change. However, their engagement with nature-related risks and opportunities remains limited. Integrating structured tools such as knowledge graphs can help financial institutions map their dependencies, identify key nature loss drivers, and align actions with frameworks like SBTN (Science Based Targets Network, 2020), while also identifying leverage points for intervention and risk mitigation across asset classes and portfolios.

Knowledge Graphs (KGs) (Ji et al., 2021) have emerged as a powerful tool to enhance LLMs by representing structured information as graphs, which can be stored in systems like the Neo4j graph database (Neo4j, 2024). KGs can help mitigate issues such as factual inaccuracies, weak reasoning, and hallucinations. By retrieving verified information from the KG, LLMs can generate more accurate queries and reduce error propagation (Ozsoy et al., 2024).

Previously, KGs have been used within the sustainability sector to help small and medium-sized enterprises report on sustainability standards. For example, OntoSustain (Zhou and Perzylo, 2023) used SPARQL queries to calculate indicators based on business activities, bringing scattered sustainability data for consistent and comparable reporting. Similarly, another study (Usmanova and Usbeck, 2024) has further built on OntoSustain by extending the KG to include ESRS 2 Topical Environmental Standards. They leveraged GPT-4 with the extended ontology to extract structured information such as strategies, risks, and climate targets and turned it into triples for KG construction. This shows how combining ontologies with large language models can support automated, standards-aligned sustainability reporting, therefore, to support complex nature-related financial decision-making, we propose a domain-specific application of LLMs and Cypher-based querying over a structured knowledge graph tailored to nature finance. By modeling nature-related dependencies, risks, and financial impacts as graph-structured data, we can enable stakeholders to interact with and extract insights from complex datasets using natural language.

Large Language Models (LLMs) have demonstrated breakthrough success in general language capabilities and question-answering tasks (Chang et al., 2024). Despite their widespread use in downstream tasks, limited work has addressed domain-specific applications. In finance, for instance, BloombergGPT (Wu et al., 2023) was proposed to enhance domain-specific knowledge in LLMs. Its performance shows that BloombergGPT outperforms other LLMs in finance-related tasks. However, adapting LLMs to highly specialized domains, such as nature finance, remains challenging due to limited domain-specific data, high adaptation costs, and persistent hallucination risks. Recent progress in financial technology highlights how artificial intelligence and distributed systems are reshaping finance. The latest review on decentralized finance (DeFi) identifies a paradigm shift in FinTech driven by blockchain-based infrastructures that enable transparent, programmable, and automated financial services (Xu et al., 2024). Similarly, emerging research on large language models in blockchain and supply-chain finance demonstrates how LLMs can enhance decision support, contract analysis, and on-chain or off-chain data integration (He et al., 2024; Srivastava et al., 2024).

This has led to a growing interest in improving text-to-Cypher conversion, which offers a graph-native alternative to SQL. Cypher is designed for querying graph-structured data and better suits domains like knowledge graphs. Several studies have focused on fine-tuning LLMs for the text-to-Cypher task. Neo4j Labs (Tomaž Bratanic, 2024) fine-tuned LLaMA and Codestral models on their datasets, while GPT4Graph (Guo et al., 2023) explored zero-shot and one-shot learning for Cypher generation. However, the performance of LLMs in zero- or few-shot text-to-Cypher tasks remains limited (Chen et al., 2021).

One of the main challenges in applying LLMs to natural language generation lies in maintaining output quality while leveraging their reasoning capabilities (Kojima et al., 2022). This paper is a proof of concept and can be seen as a foundational work to demonstrate how a KG-based framework can support financial institutions in designing better decision-making systems for nature-related information. Knowledge graphs offer a structured way to represent and analyse relationships between nature loss and financial exposure. They can help businesses and financial practitioners understand how nature loss drivers, such as land degradation, impact their portfolios. Financial institutions can incorporate this structured insight into risk assessments, investment strategies, loan evaluations, and regulatory compliance. For example, a bank financing a real estate project could assess alignment with nature-positive urban planning standards or evaluate whether a borrower's activities mitigate key drivers of biodiversity loss.

The main contributions of this paper are as follows: (1) Introduce an ontology for nature finance that defines key entities (Actions, Drivers of Nature Loss, Value Chains, Evidence, Sources) and their relationships, grounded in ENCORE and SBTN. (2) We instantiate this ontology into a knowledge graph, called NatureKG, specifically for the built environment sector. This involves combining curated expert input with LLM-assisted summarization for the Evidence nodes. (3) As a case study application, we create a Text2Cypher dataset and fine-tune open-source LLMs (Phi-3, LLaMA, Mistral) to translate natural language into Cypher queries over NatureKG. While our evaluation shows modest execution accuracy, it demonstrates feasibility and establishes a baseline for future research.

## 2 Methods

**Figure 1:**
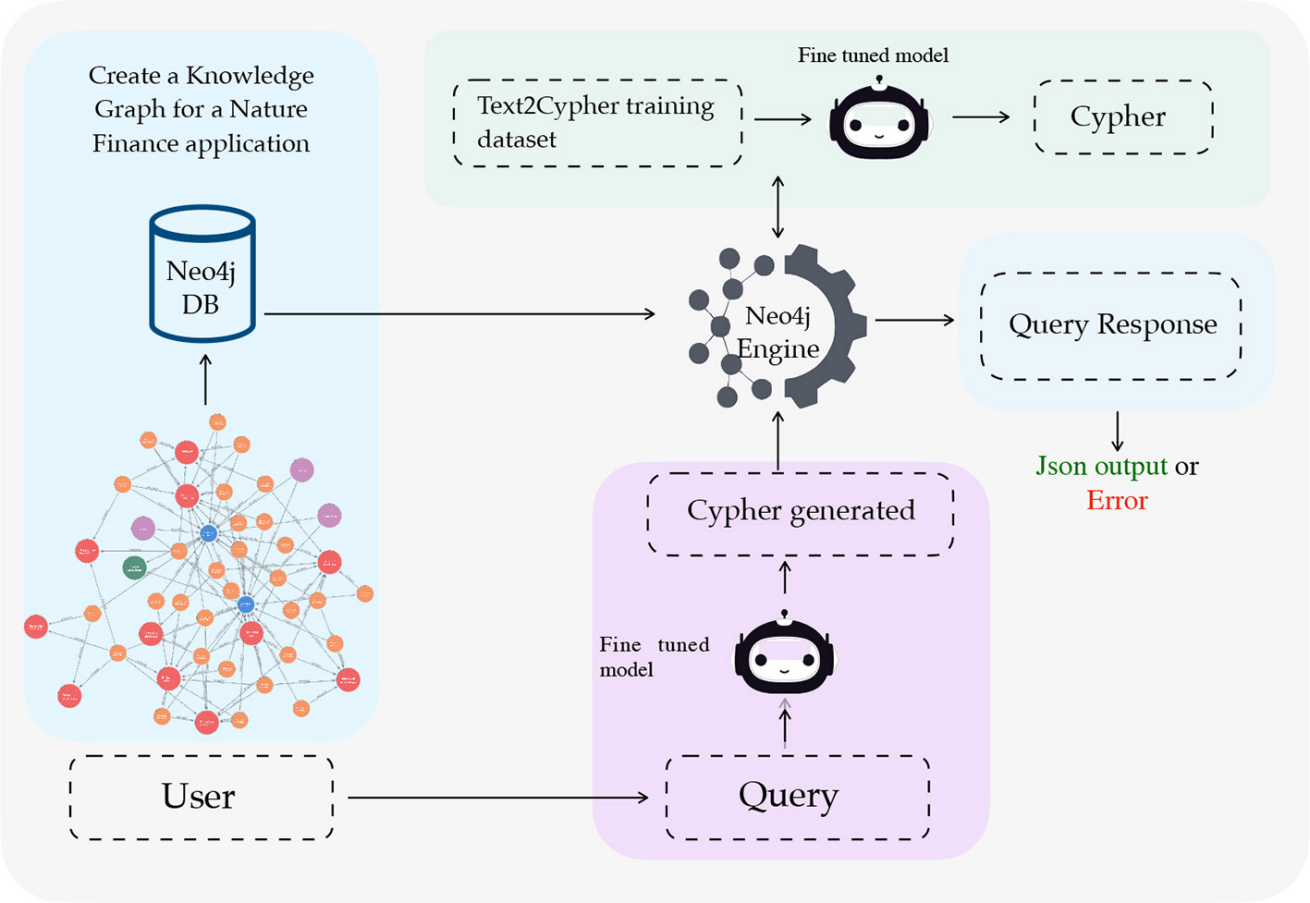
End-to-end question answering system pipeline for Text2Cypher translation for Nature Finance Knowledge Graph.

An overview of the proposed end-to-end pipeline is shown in Figure 1. The pipeline has four stages: (i) the design of an ontology that defines the core entities and relationships in nature finance, (ii) the instantiation of this ontology into a populated knowledge graph (NatureKG), (iii) the construction of a Text2Cypher dataset pairing natural language questions with Cypher queries over the KG, and (iv) a use case where large language models (LLMs) are fine-tuned to translate natural language into Cypher queries. The first two stages, ontology design and KG instantiation, constitute the primary methodological contribution of this work, while the Text2Cypher dataset and LLM fine-tuning serve as a demonstration of how NatureKG can be queried in practice.

### 2.1 Ontology design

**Figure 2:**
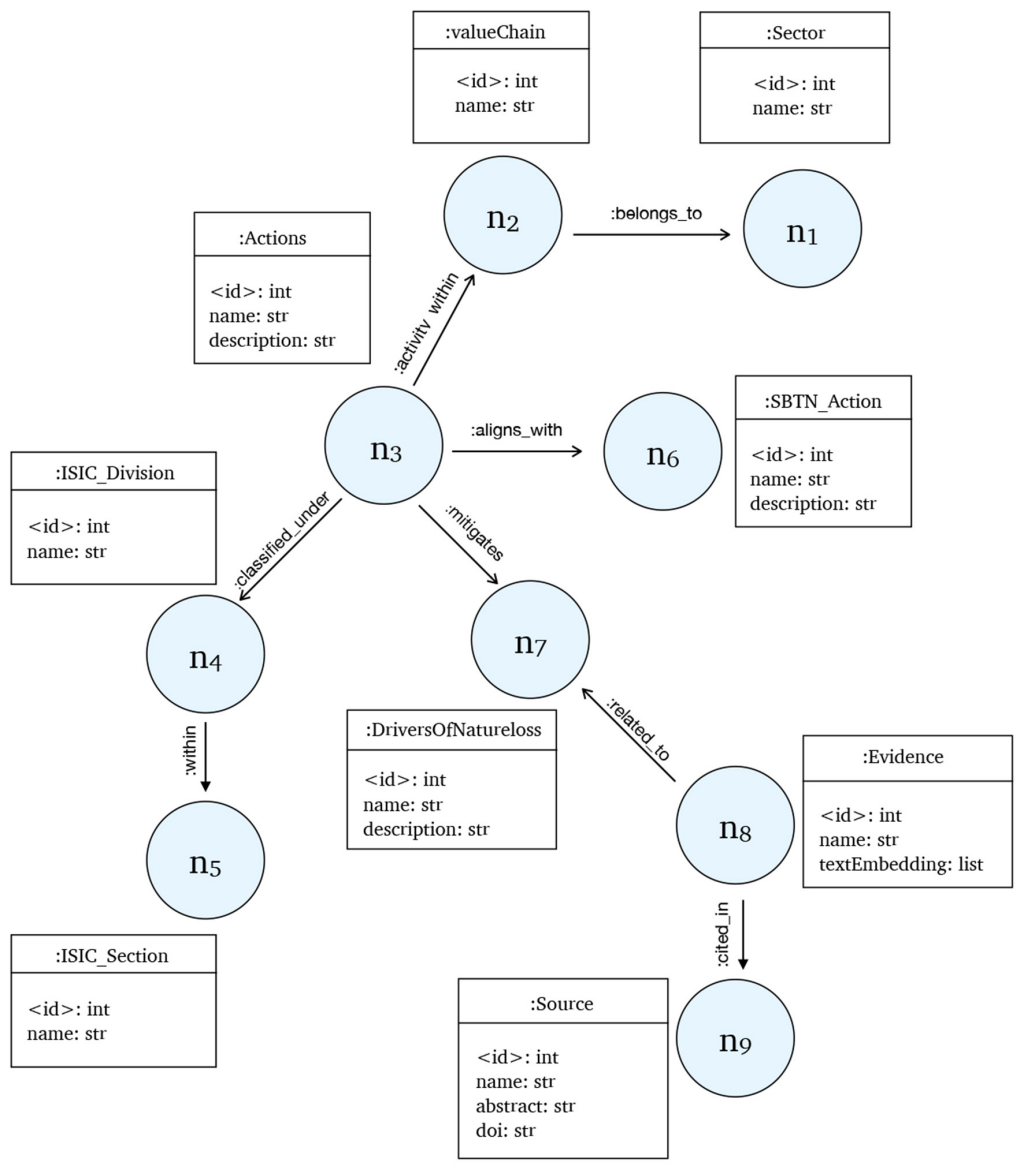
Proposed ontology for NatureKG showing nodes and their relationships. The nodes (n1– n8) refer to the node labels.

We first define an ontology that captures the domain of nature finance. The ontology specifies the classes (e.g., *Actions, DriversOfNatureLoss, ValueChain, Evidence, Sources*) and relationships (e.g., MITIGATES, CITED_IN, ALIGNS_WITH) needed to represent dependencies, risks, and interventions. This schema provides a structured foundation for reasoning about nature-related risks and opportunities. The full ontology schema is illustrated in Figure 2.

### 2.2 Knowledge graph instantiation (NatureKG)

Based on the ontology, we constructed the NatureKG knowledge graph with the help of two domain experts. Entities and relationships were extracted from authoritative sources such as ENCORE, the Science-Based Targets Network (SBTN), and academic literature. This information was curated and structured in a Neo4j database. To enrich the graph, large language models were used to generate concise summaries for *Evidence* nodes, which were subsequently validated by experts. The resulting NatureKG currently contains 320 nodes and 540 relationships.

#### 2.2.1 Data sources

To develop NatureKG, we did not rely on a single source or database. For the built environment, we utilized trusted data sources such as the ENCORE tool (Natural Capital Finance Alliance, 2023), which provides information about the thirteen environmental pressures, and the Science Based Targets Network (SBTN) (Science Based Targets Network, 2020), which offers companies guidelines and methodologies for environmental action that are both voluntary and rigorous. SBTN specifies the actions organizations must take, how much they must do, and when to protect and restore nature.

Additionally, we incorporate scientific publications, including literature, guidelines, and gray literature justifying the actions that reduce relevant pressures on nature. To ensure data quality and consistency, all collected materials were reviewed and harmonized by domain experts. Redundant or ambiguous entries were removed, and action-pressure relationships were validated against ENCORE's taxonomy of environmental pressures and the corresponding SBTN guidance. Each data record was checked for completeness and standardized terminology before integration into the Knowledge Graph schema. This information is then utilized to build the Knowledge Graph.

#### 2.2.2 Knowledge graph creation

NatureKG represents real-world entities and their relationships as nodes and edges (Table 1). Unlike traditional knowledge graphs constructed from unstructured data sources, this graph was expert-driven. Domain experts explicitly defined both the entities and relationships to ensure accuracy and relevance in the context of nature finance within the built environment sector. The graph integrates domain-specific insights to represent key elements, including value chain components, value chain stages, drivers, the SBTN action framework, actions, evidence, and sources.

**Table 1:** Node description for nature-finance knowledge graphs.

**Node label**	**Description**
Sector (n1)	Interested category of economic activity. Built environment in this case. The key property is the name of the sector.
Value chain (n2)	This identifies where the response action is applied within the value chain. The key property is the name of the value chain.
Actions (n3)	This describes the impact reduction strategy or action. It enables users to quickly understand the type of intervention. Key properties include the action name, along with its description.
International Standard Industrial Classification (ISIC) Division (n4)	ISIC Divisions are more detailed subdivisions within each ISIC section. The key property is the name of the ISIC Division.
International Standard Industrial Classification (ISIC) Section (n5)	This refers to a specific sector or sub-sector of the economy, such as crop production, mining, or manufacturing. It facilitates the analysis of pressures and responses within and across various industries, enabling sector-based comparisons and regulatory alignment. The key property is the name of the ISIC Section.
SBTN Action (n6)	The ART3 Framework typically classifies actions by type: Avoid, Reduce, Transform, and Restore. This allows for a typological grouping of nature-positive interventions. Key properties include the name of the SBTN action and its description.
Drivers of Nature loss (n7)	Shows how the action directly reduces key pressures on nature. Key properties include the type of pressure and its description.
Evidence (n8)	Provides scientifically or empirically supported justification from academic or gray literature indicating that the action reduces specific pressures on nature. Key properties of this node include relevant text, identified as the name property in the knowledge graph (KG), along with the text embedding.
Source (n9)	Contains the reference or citation of the research publication supporting the evidence node. Key properties include the title of the paper, DOI, and the abstract.

KGs represent a fact as a triplet, consisting of a subject, predicate, and object (SPO). In the field of nature finance, the actions and drivers of nature loss are the central entities. The current version of NatureKG consists of nine node types and eight defined relationship types. The nodes include Actions, ISIC_Division, DriversOfNatureLoss, ValueChain, SBTN_Action, Sector, Evidence, Source, and ISIC_Section. The relationships include CLASSIFIED_UNDER, MITIGATES, ACTIVITY_WITHIN, ALIGNS_WITH, BELONGS_TO, CITED_IN, and WITHIN. The proposed ontology graph with nodes and their relationship are shown in Figure 2. Knowledge graphs enable a variety of tasks, including semantic search, explainable AI, question answering, and information retrieval. For example, if a user asks, “What actions focus on reforestation and land restoration?” the query can be answered by retrieving the triplet (Actions, MITIGATES, DriversOfNatureLoss).

We implemented NatureKG in Neo4j (Neo4j, 2024), which was chosen for its performance, widespread adoption, and powerful querying capabilities through the Cypher language (Figure A1 in Appendix).

#### 2.2.3 Built environment dataset

The underlying dataset of impact reduction strategies for the built environment sector was developed to support the integration of actionable response options into NatureKG. This dataset is designed to help companies and financial institutions (for businesses in their lending portfolio) to identify practical ways to change business practices to address biodiversity and ecosystem services considerations across high-risk activities. It aligns with the SBTN mitigation hierarchy of Assess, Commit, Transform, and Disclose (ACT-D) (Science Based Targets Network, 2020), and with the World Business Council for Sustainable Development (WBCSD) (World Business Council for Sustainable Development, 2021). The dataset analyses options to mitigate impacts on nature, including those resulting from the thirteen pressures identified by the ENCORE tool (Natural Capital Finance Alliance, 2023).

In this use case of NatureKG, the underlying data for the built environment has been curated to ensure replicability. This dataset grounds the ontology in a real-world sector, demonstrating its practical applicability.

### 2.3 Text2Cypher dataset construction

After the construction of the NatureKG to perform nature language queries using LLMs, a domain specific Text2Cypher dataset was built for LLMs instruction fine-tuning with the help of a domain expert. Each cypher query is validated for syntax errors by running it in a local Neo4j graph database of NatureKG. Cypher queries that cause errors are corrected, and duplicate entries are removed from the Text2Cypher dataset to get unique (question, cypher) pairs. Our NatureKG currently focuses on one sector: Built Environment. NatureKG consists of eight node types and nine relationship types, resulting in 320 nodes and 540 relationships. To enable effective fine-tuning of LLMs on this relatively small graph, we performed data augmentation by paraphrasing questions associated with the same or similar cypher queries (keeping the uniqueness and complexity of the query) to expand the dataset. In addition to standard cypher queries that define pattern-based conditions, the dataset also contains several queries that make use of regular expressions.

## 3 Experimental setup and results

### 3.1 Dataset split strategies and experimental setup

The LLMs were fine-tuned using Text2Cypher dataset, which consists of 545 samples. To evaluate linguistic and partial structural generalization of the NatureKG model, we employed dataset-splitting strategies: paraphrase-level splitting, cypher-level splitting, and schema component generalization. The datasets were split into 80:20 for training and testing. There are 455 training samples and 109 testing samples for three splits, but the paraphrasing split consists of 455 and 90, respectively. The split strategies are described as follows:

**Paraphrasing split:** In this criterion, we group all paraphrases corresponding to the same cypher query and assign them exclusively to either the training or test set (Finegan-Dollak et al., 2018) as shown in Table 2. Within each group, we randomly sample 10–20% of the paraphrases to the test set. This method allows the model to encounter unseen linguistic variations during testing that it has not seen during training. However, this setup does not assess the model's ability to handle structurally novel cypher queries or previously unseen combinations of graph elements, such as relations or node types.

**Table 2:** Example of paraphrase-based split for training and testing.

**Type**	**Content**
Cypher	MATCH (a:Actions)-[:MITIGATES]->(d:DriversOfNatureLoss) WHERE toLower(d.name) = ‘emissions of ghg' RETURN a.name, d.name
Training	Show actions addressing GHG emissions.
Testing	Which actions help mitigate emissions of GHG?

**Cypher-Level Split:** In this approach, natural language paraphrases that correspond to the same underlying cypher query are grouped together and assigned exclusively to either the training set or the test set (Finegan-Dollak et al., 2018) as shown in Table 3. It is done by extracting schema components (i.e., node and relation types) from each query and grouping all rows that share the same structure. The groups are shuffled and added to the test set until it reaches 20% of the dataset, with the remaining groups allocated to the training set. This ensures that no exact same cypher query appears in both splits and preserving evaluation integrity at the query structure level. It tests the model's ability for linguistic generalize.

**Table 3:** Example of Cypher split for training and testing.

**Split**	**Question and corresponding cypher query**
Training	*Show actions within construction that help limit GHG emissions*.
	Cypher:
	MATCH (a:Actions)-[:MITIGATES]->(d:DriversOfNatureLoss),
	(a)-[:ACTIVITY_WITHIN]->(v:valueChain)
	WHERE toLower(d.name) = ‘emissions of ghg'
	AND toLower(v.name) CONTAINS ‘construction'
	RETURN a.name, d.name, v.name
**Testing**	*What are effective solutions to mitigate GHG emissions in the construction stage?*
	**Cypher:**
	MATCH (a:Actions)-[:MITIGATES]->(d:DriversOfNatureLoss),
	(a)-[:ACTIVITY_WITHIN]->(v:valueChain)
	WHERE d.name = ‘(?i).*emissions of ghg.*'
	AND v.name = ‘(?i).*construction.*'
	RETURN a.name, d.name, v.name

Generalization split: In this approach, we divide the dataset based on the schema components used in the cypher queries (Yu et al., 2018). First, we defined the schema, which includes all nodes and relevant relationship types (e.g., Actions, DriversOfNatureLoss, MITIGATES, ALIGNS_WITH, etc.). For each query, we identified the unique set of schema elements it used and grouped queries that shared the same node-relationship combinations (Table 4). Similar to paraphrasing and cypher-level split set creation, we generated the test and training sets. Our evaluation focuses on two aspects: (1) Schema Transferability: assesses whether training on one portion of the schema improves the model's ability to generate accurate queries for other, unseen parts; and (2) Structural generalization: evaluation of the model's ability to generalize unseen subgraphs, relationships, and node types.

**Table 4:** Examples of generalization in the test set with unseen schema combinations.

**Split**	**Query (NL)**	**Cypher query (truncated)**	**Schema components**
Train	Which actions mitigate water use?	MATCH (a)-[:MITIGATES]->(d)	{Actions, DriversOfNatureLoss, MITIGATES}
Test	Find actions that align with SBTN principles to mitigate water use.	MATCH (a)-[:MITIGATES]->(d), (a)-[:ALIGNS_WITH]->(s)	{Actions, DriversOfNatureLoss, MITIGATES, ALIGNS_WITH, SBTN_Action}
Train	Which actions reduce nature loss drivers?	MATCH (a)-[:MITIGATES]->(d)	{Actions, DriversOfNatureLoss, MITIGATES}
Test	What sources cite research on solutions for disturbances?	MATCH (e)-[:RELATED_TO]->(d), (e)-[:CITED_IN]->(s)	{Evidence, DriversOfNatureLoss, CITED_IN, Source, RELATED_TO}

There is a potential risk that similar natural-language questions linked to the same Cypher query could appear across splits and lead to memorization since the dataset was expanded through paraphrasing. To minimize this, all paraphrased variants of a given Cypher query are grouped within a single split so that no question-Cypher pair is shared between training and test data. This grouping prevents lexical overlap between splits and ensures that the model cannot recall previously seen Cypher templates. The Cypher-Level and Generalization splits were specifically designed to evaluate higher-order generalization while mitigating any remaining risk of memorization from paraphrased data. For the Cypher-Level split, we excluded every Cypher template that appeared in training so that the test set contained only new query structures. The Generalization split went a step further by also removing unseen combinations of nodes and relationships, testing both structural and semantic generalization. Using both splits helps reduce any memorization that could result from paraphrased data and gives a clearer picture of how the model performs on unseen cases.

Experimental setup: We fine-tuned three instruction-tuned language models: LLaMA 3 (8B Instruct), Mistral (7B), and Phi-3 using LoRA (Hu et al., 2022) with 4-bit quantization (via bitsandbytes) to reduce memory usage. All models were trained for 2 epochs with a batch size of 2 and gradient accumulation of 4, using the AdamW optimiser (8 bit), a learning rate of 2e-4, linear scheduler, and bf16 precision. All experiments were conducted using two A100 GPUs. The training on two A100 GPUs was challenging due to limited computational power, which was addressed by using a quantization approach.

### 3.2 Evaluation metrics

The performance of all the models is evaluated using different evaluation metrics, including the BLEU score (Papineni et al., 2002), exact match, execution accuracy and Cypher Difficulty Criteria (Yu et al., 2018).

#### 3.2.1 BLEU and exact match

The BLEU score is a translation-based metric to evaluate the syntactic similarity between the predicted Cypher queries and the ground truth Cypher queries. BLEU is sensitive to ngram overlap between predicted and reference queries and does not capture semantic meaning. Exact Match accuracy determines whether the predicted Cypher query is semantically equivalent to the ground truth. Furthermore, we conducted a clause-level evaluation to provide a more detailed analysis of model performance. Specifically, we computed exact set matching *F1 score* between the predicted and ground truth Cypher queries across individual components (Yu et al., 2018): MATCH, WHERE, and RETURN clauses. We reported the Macro *F*1*score* for each evaluation split, averaged across these components. A high MATCH *F1* shows that the model can correctly identify graph structures, WHERE clause *F1* show how well the model can apply logical filtering conditions, and the RETURN clause *F1* measures the correctness of the output variables or entities retrieved from the graph.

#### 3.2.2 Execution-based accuracy

To validate the cypher generated by the fine-tuned LLM model, we executed it on the neo4j graph database. During execution, we identified three outcomes:

1) The query executes successfully and returns results.

2) An error occurs, and we store the type of error

3) The query executes successfully, but the returned result is None. To report these results, we calculated four metrics: Error Rate: the proportion of queries that failed to run; Execution Rate: the percentage of queries that are executed without errors; ExecAcc: the exact match between the ground truth and the predicted value; and Partial ExecAcc: partial match between the ground truth and the predicted value results.

#### 3.2.3 Cypher difficulty criteria

We also measure the model performance as a function of the difficulty of the cypher query. We categorized these queries into three difficulty levels- easy, medium, and hard to better understand the performance (Table 5). These difficulty levels are defined based on components of cypher queries such as MATCH clause and WHERE filters: (1) Easy: Queries that contain a single pattern traversal and no filter conditions. (2) Medium: Queries that contain only a single pattern traversal and include up to two simple filter conditions in the WHERE clause, typically joined by logical operators such as AND, OR, or commas. (3) Hard: Queries that contain only a single pattern traversal but include more than two filter conditions. In our implementation, the number of filters is determined by splitting the WHERE clause using logical operators such as AND, OR, and commas.

**Table 5:** Examples of cypher queries by difficulty level.

**Difficulty level**	**Example**
**Split**	**Question and corresponding cypher query**
**Easy**	**Input:** *List all actions and their names?*
	**Cypher:** MATCH (a:Actions) RETURN a.name
**Medium**	**Input:** *What strategies mitigate the driver of freshwater use?*
	**Cypher:** MATCH (a:Actions)-[:MITIGATES]->(d:DriversOfNatureLoss) WHERE toLower(d.name) CONTAINS ‘freshwater' RETURN a.name, d.name
**Hard**	**Input:** *Which actions deal with biotic resource extraction impacts?*
	**Cypher:** MATCH (a:Actions)-[:MITIGATES]->(d:DriversOfNatureLoss) WHERE toLower(d.name) CONTAINS ‘biotic' AND toLower(d.name) CONTAINS ‘resource' AND toLower(d.name) CONTAINS ‘extraction' AND NOT toLower(d.name) CONTAINS ‘abiotic' RETURN a.name, d.name

An example of a cypher query for all difficulty levels is shown in Table 5. We evaluate model performance at each difficulty level using exact match accuracy, BLEU score, and clause-level F1 metrics (MATCH, WHERE, RETURN) to evaluate detailed prediction accuracy.

## 4 Discussion

### 4.1 Model finetuning and evaluation

We fine-tuned three open-source LLMs for the task of Text2Cypher within a domain-specific NatureKG. The instruction versions of these models are Phi-3-medium-4k-instruct (Abdin et al., 2024), llama-3-8b-instruct (Grattafiori et al., 2024), and mistral-7b-instruct-v0.2-bnb-4bit (Mistral AI, 2023). These models were selected by considering both technical and practical feasibility. All three are open-source instruction-tuned models that can be fine-tuned efficiently with limited computational resources. LLM models were instruction fine-tuned using Unsloth (Unsloth, 2024) interfaces on GPU machines. Each model utilized different instruction-style prompt templates, examples of which are illustrated in Figures A3–A5 in Appendix, respectively, and the graph schema used is shown in Figure A2 in Appendix. The summary of the Prompt format structure used during fine-tuning across different models is shown in Table 6.

**Table 6:** Prompt format structure for LLM models.

**Model**	**Prompt format summary**
Phi-3 (Unsloth)	Plain instruction-input-response format with EOS token
LLaMA-3 (Unsloth)	Uses < |user|> and < |assistant|> tags, includes headers like ### Instruction
Mistral-7B (Unsloth)	ChatML-style prompts with < |im_start|> and < |im_end|> role blocks

The evaluation results for our model's performance are based on various metrics, including BLEU, Exact Match (EM), error rate, execution rate, execution accuracy (ExecAcc), and partial execution accuracy (Table 7). Phi-3 achieves the highest execution accuracy on paraphrase splits (0.21), with consistently high execution accuracy on generlarization (0.14) and partial match scores, peaking at 0.60 on the Cypher-level split. LLaMA-3 8B performs moderately well, with high execution rates (0.981) and competitive partial match scores (0.60) under schema generalization. In comparison, Mistral-7B achieves near-zero execution accuracy and partial scores across all splits.

**Table 7:** Evaluation results.

**Model**	**Split**	**BLEU**	**Exact match (%)**	**Error rate**	**Execution rate**	**ExecAcc**	**Partial ExecAcc**
**Phi-3**	**Paraphrase**	**0.305**	**10.0**	**0.023**	**0.977**	**0.21**	**0.56**
	Cypher-Level	**0.186**	**1.8**	0.090	0.910	**0.09**	**0.60**
	Generalization	**0.238**	**6.4**	0.112	0.888	**0.14**	0.39
**LLaMA-3 8B**	Paraphrase	0.264	6.7	0.059	0.941	0.13	0.47
	Cypher-Level	0.165	**1.8**	**0.028**	**0.972**	0.06	0.51
	Generalization	0.212	5.5	**0.019**	**0.981**	0.12	**0.60**
**Mistral-7B**	Paraphrase	0.203	0.0	0.956	0.044	0.0	0.01
	Cypher-Level	0.121	0.0	0.982	0.018	0.0	0.0
	Generalization	0.146	0.0	0.954	0.046	0.0	0.0

Bold values indicate the highest score achieved within each evaluation split for that metric.

Additionally, the clause-level evaluation presented in Table 8 shows that Phi-3 achieves the highest MACRO_F1 scores across all splits, particularly excelling in the RETURN clause performance, reaching up to 0.83 on the Paraphrase split. The LLaMA-3 8B model demonstrates balanced performance across different clauses, especially in the Paraphrase split. In contrast, Mistral-7B consistently underperforms, showing low scores in both RETURN and MACRO_F1. However, it displays relatively higher performance for MATCH clause in the generalization split (scoring 0.60).

**Table 8:** Clause-level F1 scores for Cypher generation (MATCH, WHERE, RETURN, MACRO_F1) across evaluation splits.

**Model**	**Split**	**MATCH**	**WHERE**	**RETURN**	**MACRO_F1**
**Phi-3 (4k)**	Paraphrase	**0.72**	0.12	**0.83**	**0.56**
	Cypher-Level	**0.49**	**0.10**	0.64	**0.41**
	Generalization	0.57	0.13	0.72	**0.47**
**LLaMA-3 8B**	Paraphrase	0.62	0.12	0.78	0.51
	Cypher-Level	0.38	**0.10**	0.67	0.38
	Generalization	0.46	**0.17**	0.69	0.44
**Mistral-7B**	Paraphrase	0.57	**0.13**	0.30	0.33
	Cypher-Level	0.39	0.08	0.21	0.22
	Generalization	**0.60**	0.06	0.38	0.35

Bold values indicate the highest score achieved within each evaluation split for that metric.

**Figure 3:**
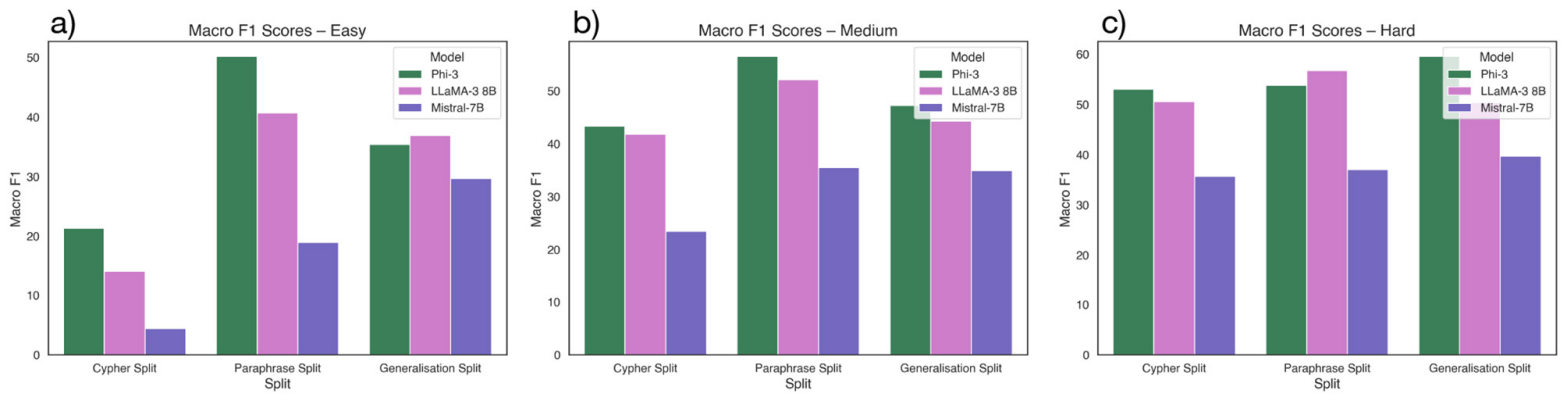
Macro F1 scores of models across Cypher, Paraphrase, and generalization splits, shown separately for **(a)** Easy, **(b)** Medium, and **(c)** Hard difficulty levels.

Across all difficulty levels, Phi-3 consistently achieves the highest performance, particularly in structural metrics like MATCH, RETURN, and Macro F1. It performs best on the Paraphrase split, reaching a Macro F1 of 50.18% (Easy), 56.51% (Medium), and 59.57% (Hard) as shown in Figure 3 and Tables A1–A3 in Appendix. LLaMA-3 8B shows competitive and balanced performance, especially under paraphrased inputs, while Mistral-7B lags across most metrics and struggles with generalization.

All fine-tuned models show the ability to predict correct cypher both syntactically and semantically. These results should be viewed as a baseline feasibility study that shows that smaller models such as Phi-3 can sometimes generalize better than larger ones in low-resource, domain-specific settings.

However, during our analysis, we identified some common error patterns in the fine-tuned models. First, we observed a naming mismatch, where the model predicted a correct structure but used slightly aliased names when returning the results. It is observed that such errors arise from semantic ambiguity in natural language, particularly where domain-specific terminology has multiple interpretations. For example, the term “pressure” in nature research refers to drivers or factors contributing to nature loss, whereas in general contexts it denotes different meanings, such as economic pressure or market stress. Such contextual ambiguity can lead the LLM model to select incorrect nodes or relationships within the knowledge graph. Second, the model tends to introduce additional fields that are not present in the ground truth cypher query. Third, the models confuse node labels with node properties, either generating node types in place of properties or misidentifying the correct property names. Fourth, models frequently struggle to determine when to use exact matches versus partial matches in property conditions, for example, choosing between = and CONTAINS.

### 4.2 Limitations

Our proposed NatureKG is focused only on the built environment sector, which results in fewer nodes and relationships. Consequently, we were unable to generate more training data. The paraphrasing technique is used for questions to create more training examples that contain the same or similar cypher queries, which may result in memorization of cypher output during training of fine-tuned models.

While our method evaluates linguistic generalization, it does not capture the model's ability to handle structurally novel cypher queries or previously unseen combinations of graph elements such as relationships or node types. The setup focuses on linguistic generalization instead of structural generalization, as the KG schema remains fixed within the nature finance domain, it effectively measures the model's ability to adapt to variations in user intent.

## 5 Conclusion

In this work, we introduced NatureKG, the first ontology and knowledge graph specifically tailored to the nature finance domain. While the purpose of the knowledge graph is to ensure objective accuracy, we acknowledge that it may not be entirely exhaustive and some gaps may still exist. The ontology and its instantiation as NatureKG provide a structured foundation for representing risks, dependencies, and interventions, and can serve as a reusable resource for research and applications. As a proof of concept, we developed a Text2Cypher dataset and fine-tuned LLMs to query NatureKG. We agree that even modest inaccuracies in the model can be consequential in financial decision-support systems. Therefore, reported models' performance should be interpreted as a baseline. The experimental results show that among all three fine-tuned LLMs, Phi-3 achieved the highest execution accuracy (0.21) and Macro F1 (0.56), demonstrating better structural and reasoning capability, while LLaMA-3.1 8B showed balanced performance across evaluation splits. Clause-level analysis revealed that models perform well in identifying graph structures and outputs but face challenges in logical reasoning, indicating the need for further domain-specific fine-tuning and data expansion. The paper's goal is to establish the feasibility of integrating domain-specific ontologies with LLMs under limited data conditions. In practical settings, acceptable thresholds for query accuracy will depend on use-case sensitivity and should be explored in future work.

## Data Availability

Publicly available datasets were analyzed in this study. This data can be found here: https://zenodo.org/records/16965298.
