# Supplementary material for: NatureKG: an ontology and knowledge graph for nature finance with a Text2Cypher application
Source: Front Artif Intell. 2025 Dec 4;8:1693843. doi: 10.3389/frai.2025.1693843 (PMC12713199; doi:10.3389/frai.2025.1693843)
Supplement: Supplementary file 1 [file Data_Sheet_1.pdf]

A Appendix

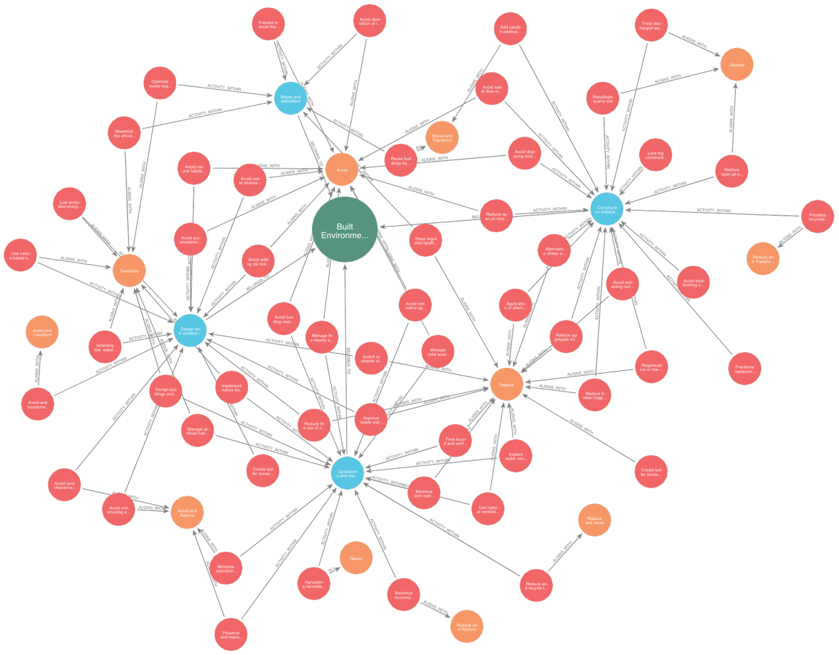

Figure A1: Knowledge Graph for Nature-aligned activities in the Built-Environment. Green: Sector, Blue: Value chain stages, Orange: SBTN mitigation hierarchy, Red: Impact reduction strategies.

```
Node properties:
Sector
name: STRING Example: ['Built Environment’]
Actions
name: STRING Example: "Avoid non native species plantation”
description: STRING
valueChain
name: STRING Examples: ['Construction materials and mineral extraction for
all urban infrastructure', 'Design and construction', 'Operations and
maintenance', 'Waste and demolition’]

SBTN_Action
name: STRING Example: "Reduce"
description: STRING Example: "Take steps to minimise any unavoidable impacts
on nature. This involves reducing the intensity and extent of negative
effects that cannot be entirely avoided.”

DriversOfNatureLoss
name: STRING Example: "Disturbances"
description: STRING Example: "Activity produces noise or light pollution that
has potential to harm organisms…”

Source
name: STRING Example: "Kunz, N.C. (2020) ‘Towards a broadened view of water
security in mining regions’, Water Security, 11, p. 100079. doi:10.1016/
j.wasec.2020.100079."
abstract: STRING
doi: STRING Example: "doi: https://doi.org/10.1016/j.wasec.2020.100079"
title: STRING Example: "title: Towards a broadened view of water security in
mining regions”

Evidence
name: STRING Example: "Noise pollution have caused animals to flee”

ISIC_Section
name: STRING Example: "Mining and Quarrying”

ISIC_Division
name: STRING Example: "Quarrying of stone, sand and clay”

The relationships:
(:Actions)-[:CLASSIFIED_UNDER]->(:ISIC_Division)
(:Actions)-[:MITIGATES]->(:DriversOfNatureLoss)
(:Actions)-[:ACTIVITY_WITHIN]->(:valueChain)
(:Actions)-[:ALIGNS_WITH]->(:SBTN_Action)
(:valueChain)-[:BELONGS_TO]->(:Sector)
(:Evidence)-[:RELATED_TO]->(:DriversOfNatureLoss)
(:Evidence)-[:CITED_IN]->(:Source)
(:ISIC_Division)-[:WITHIN]->(:ISIC_Section)
```

Figure A2: Graph schema for NatureKG.

```
<|user|>
### Instruction:
Use the below text to generate a Cypher query. The schema is given below:
```{Graph schema}```
### Input:
Which value chains are involved in mitigating emission of nutrient pollutants
to water?
<|assistant|>
MATCH (a:Actions)-[:MITIGATES]->(d:DriversOfNatureLoss), (a)-
[:ACTIVITY_WITHIN]->(v:valueChain)
WHERE toLower(d.name) CONTAINS 'emission' AND toLower(d.name) CONTAINS
'nutrient'
AND toLower(d.name) CONTAINS 'pollutants' AND toLower(d.name) CONTAINS 'water'
RETURN DISTINCT d.name, v.name
```

Figure A3: Prompt format used for instruction fine-tuning with LLaMA-3 8B model.

```
Given are the instructions below, along with an input that provides further
context.
### Instruction:
Use the text below to generate a Cypher query. The schema is given below:
```{Graph schema}```
### Input:
Which value chains are involved in mitigating emission of nutrient pollutants
to water?
### Response:
MATCH (a:Actions)-[:MITIGATES]->(d:DriversOfNatureLoss), (a)-
[:ACTIVITY_WITHIN]->(v:valueChain)
WHERE toLower(d.name) CONTAINS 'emission' AND toLower(d.name) CONTAINS
'nutrient'
AND toLower(d.name) CONTAINS 'pollutants' AND toLower(d.name) CONTAINS 'water'
RETURN DISTINCT d.name, v.name
```

Figure A4: Prompt format used for instruction fine-tuning with Phi-3 (4k) model.

```
<|im_start|>system
Use the below text to generate a Cypher query. The schema is given below:
```{Graph schema}```
<|im_end|>
<|im_start|>user
Which value chains are involved in mitigating emission of nutrient pollutants
to water?
<|im_end|>
<|im_start|>assistant
MATCH (a:Actions)-[:MITIGATES]->(d:DriversOfNatureLoss), (a)-
[:ACTIVITY_WITHIN]->(v:valueChain)
WHERE toLower(d.name) CONTAINS 'emission' AND toLower(d.name) CONTAINS
'nutrient'
AND toLower(d.name) CONTAINS 'pollutants' AND toLower(d.name) CONTAINS 'water'
RETURN DISTINCT d.name, v.name<|im_end|>
```

Figure A5: Prompt format used for instruction fine-tuning with Mistral-7B model.
```

Table A1: Evaluation for difficulty level: Easy

Split	Model	EM	BLEU	MATCH	RETURN	Macro F1
Cypher Split	Phi-3 (4k)	0.00%	**20.58%**	**38.10%**	25.87%	**21.32%**
	LLaMA-3 8B	**4.76%**	11.58%	4.76%	**37.62%**	14.13%
	Mistral-7B	0.00%	3.39%	0.00%	13.17%	4.39%
Paraphrase Split	Phi-3 (4k)	**61.54%**	**65.27%**	**69.23%**	**81.32%**	**50.18%**
	LLaMA-3 8B	46.15%	50.86%	53.85%	68.21%	40.68%
	Mistral-7B	0.00%	19.29%	30.77%	26.15%	18.97%
generalization Split	Phi-3 (4k)	**43.75%**	**45.44%**	**47.92%**	58.29%	35.40%
	LLaMA-3 8B	37.50%	40.01%	45.83%	**64.91%**	**36.91%**
	Mistral-7B	0.00%	26.85%	**47.92%**	41.16%	29.69%

Table A2: Evaluation for difficulty level: Medium

Split	Model	EM	BLEU	MATCH	WHERE	RETURN	Macro F1
Cypher Split	Phi-3 (4k)	**3.13%**	**20.20%**	**47.86%**	**11.59%**	70.54%	**43.33%**
	LLaMA-3 8B	1.56%	15.85%	44.79%	5.99%	**74.57%**	41.78%
	Mistral-7B	0.00%	13.05%	43.07%	6.98%	20.31%	23.45%
Paraphrase Split	Phi-3 (4k)	**1.61%**	**25.98%**	**72.53%**	**13.12%**	**83.89%**	**56.51%**
	LLaMA-3 8B	0.00%	22.59%	63.71%	12.58%	79.94%	52.08%
	Mistral-7B	0.00%	20.99%	63.76%	12.90%	29.73%	35.47%
generalization Split	Phi-3 (4k)	0.00%	**20.50%**	**56.23%**	14.07%	**71.39%**	**47.23%**
	LLaMA-3 8B	0.00%	17.43%	45.11%	**18.66%**	68.99%	44.25%
	Mistral-7B	0.00%	11.13%	61.90%	4.11%	38.70%	34.91%

Table A3: Evaluation for difficulty level: Hard

Split	Model	EM	BLEU	MATCH	WHERE	RETURN	Macro F1
Cypher Split	Phi-3 (4k)	0.00%	12.50%	**62.50%**	15.73%	**80.83%**	**53.02%**
	LLaMA-3 8B	0.00%	**22.59%**	49.31%	**29.63%**	72.78%	50.57%
	Mistral-7B	0.00%	17.06%	60.42%	18.21%	28.33%	35.65%
Paraphrase Split	Phi-3 (4k)	0.00%	**20.71%**	61.11%	20.25%	79.97%	53.78%
	LLaMA-3 8B	0.00%	19.24%	**70.44%**	18.89%	**80.89%**	**56.74%**
	Mistral-7B	0.00%	18.63%	52.67%	**26.10%**	32.22%	36.99%
generalization Split	Phi-3 (4k)	0.00%	18.15%	**71.88%**	20.45%	**86.38%**	**59.57%**
	LLaMA-3 8B	0.00%	**20.42%**	52.08%	**23.04%**	75.94%	50.35%
	Mistral-7B	0.00%	19.34%	64.58%	22.38%	32.08%	39.68%
